# Chloroplasts: state of research and practical applications of plastome sequencing

**DOI:** 10.1007/s00425-016-2551-1

**Published:** 2016-06-03

**Authors:** Szymon Adam Olejniczak, Ewelina Łojewska, Tomasz Kowalczyk, Tomasz Sakowicz

**Affiliations:** Department of Genetics and Plant Molecular Biology and Biotechnology, The University of Lodz, Banacha Street 12/16, 90-237 Lodz, Poland

## Abstract

**This review presents origins, structure and expression of chloroplast genomes. It also describes their sequencing, analysis and modification, focusing on potential practical uses and biggest challenges of chloroplast genome modification.**

During the evolution of eukaryotes, cyanobacteria are believed to have merged with host heterotrophic cell. Afterward, most of cyanobacterial genes from cyanobacteria were transferred to cell nucleus or lost in the process of endosymbiosis. As a result of these changes, a primary plastid was established. Nowadays, plastid genome (plastome) is almost always circular, has a size of 100–200 kbp (120–160 in land plants), and harbors 100–120 highly conserved unique genes. Plastids have their own gene expression system, which is similar to one of their cyanobacterial ancestors. Two different polymerases, plastid-derived PEP and nucleus-derived NEP, participate in transcription. Translation is similar to the one observed in cyanobacteria, but it also utilizes protein translation factors and positive regulatory mRNA elements absent from bacteria. Plastoms play an important role in genetic transformation. Transgenes are introduced into them either via gene gun (in undamaged tissues) or polyethylene glycol treatment (when protoplasts are targeted). Antibiotic resistance markers are the most common tool used for selection of transformed plants. In recent years, plastome transformation emerged as a promising alternative to nuclear transformation because of (1) high yield of target protein, (2) removing the risk of outcrossing with weeds, (3) lack of silencing mechanisms, and (4) ability to engineer the entire metabolic pathways rather than single gene traits. Currently, the main directions of such research regard: developing efficient enzyme, vaccine antigen, and biopharmaceutical protein production methods in plant cells and improving crops by increasing their resistance to a wide array of biotic and abiotic stresses. Because of that, the detailed knowledge of plastome structure and mechanism of functioning started to play a major role.

## Introduction: origin of chloroplasts

**Fig. 1:**
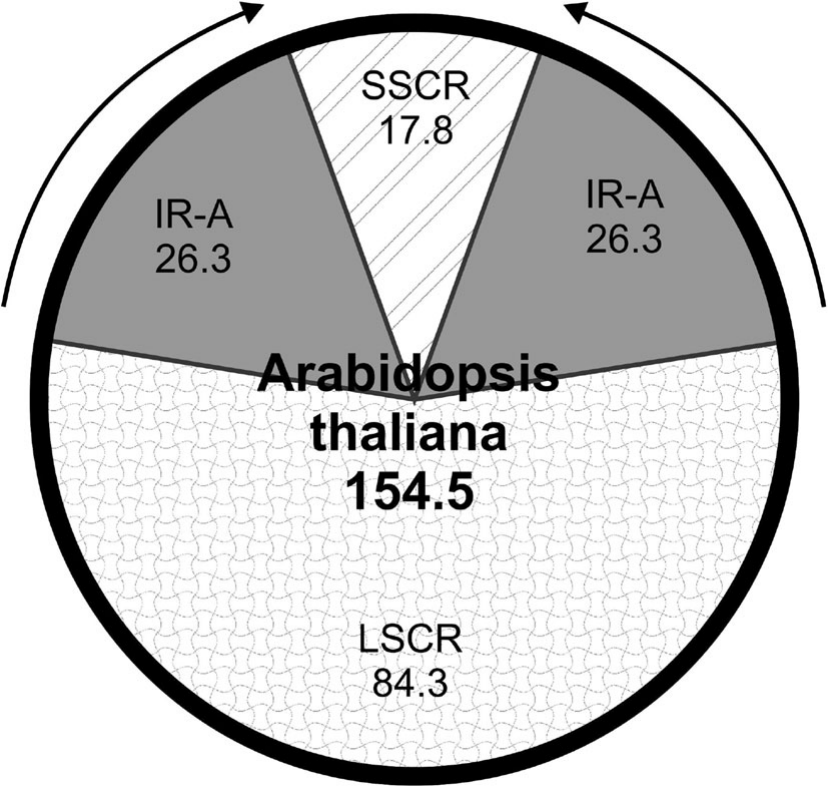
Structure of a typical chloroplast genome on example of *Arabidopsis thaliana*. *SSCR* small single copy region, *LSCR* large single copy region, and *IR-A and IR-B* inverted repeats. Length of plastome and its parts in kbp

Organelles known as chloroplasts are characteristic for plant cells and eukaryotic algae (Leister 2003). Their main purpose is housing numerous metabolic reactions necessary for the life of the cell, such as photosynthesis—production of nutrients from water and carbon dioxide with use of absorbed sunlight. They are believed to have originated from cyanobacteria, which have either invaded or been engulfed by a heterotrophic host cell approximately 1.5 billion years ago (Chan et al. 2011). In the process of primary endosymbiogenesis, the vast majority of cyanobacterial genes has either been lost or transferred to nucleus of host cell. In parallel to these changes, some host genes acquired leading sequences, which made the transport of their products into the organelle possible (Green 2011). These changes eventually led to establishment of organelle known as primary plastid (Fig. 1). However, endosymbiosis has not ended there. Majority of species belonging to Plantae has acquired secondary chloroplasts by establishing an endosymbiotic connection with chloroplast-containing algae. The process begins with engulfment (or invasion by) a plastid-containing algal cell, which eventually either loses its nuclear genes or transfers them to the nucleus of a host cell (Fig. 1) (Green 2011; Turmel et al. 2009). It is worth noting that organelle-to-host nucleus gene transfer continues to this day (Kleine et al. 2009). This review will focus on analysis of the current knowledge about structure of chloroplast genomes and potential practical applications of plastome sequencing and engineering. The terms ‘plastid’ and ‘chloroplast’ will be used throughout the text interchangeably. The former term regards plastids that are not capable of photosynthesis due to the lack of chlorophyll.

## Structure of chloroplast genome

Each plastid has its own independent genome. The vast majority of analyzed plastid DNA molecules (cpDNA) is inherited maternally and was found to be double-stranded and have a circular structure. However, it is worth noting that branched and linear cpDNA molecules may also exist in some plants, such as maize seedlings and *Medicago truncatula* (Shaver et al. 2008, Oldenburg and Bendich 2004). cpDNA molecules are a part of protein-DNA complex known as nucleoid. A single nucleoid contains 10–20 cpDNA copies and may be (1) surrounded by thylakoids in the center of chloroplast (red algae), (2) found in circular form in girdle lamellae (brown algae), or (3) found in matrix, between thylakoids (Kuroiwa 1991, Kobayashi et al. 2002). cpDNA usually exists in form of a monomer, but it is also capable of creating multimers (dimers, trimers, and the least common tetramers) (Lu et al. 2011). Unlike nuclear genome, plastome does not create complexes with histones. However, the presence of plastome-encoded, histone-like HC protein has been confirmed in red algae (Kobayashi et al. 2002).

200–300 identical DNA molecules exist in the chloroplast. Plastome size usually varies from 100 to 200 kbp. The biggest size variation can be observed in green algae chloroplast genomes. The smallest ones have been identified in *Helicosporidium* sp. *Simulium jonesie*—37.4 kbp and *Ostreococcus tauri* (71.6 kbp), while the largest ones were found in *Nephroselmis olivacea* (200.8 kbp) and *Dunaliella salina* (269 kbp) (Smith et al. 2010). Such differences have not been observed among land plants—their plastome size usually is within 120–160 kbp. (Table 1).

**Table 1:** Examples of selected parameters of the plastomes in different species

Species	Sequence length (kbp)				Number of genes		Number of introns
	Total plastome	IR (A and B)	SSC	LSC	Protein	RNA	
*Arabidopsis thaliana*	154.5	26.3	17.8	84.2	85	44	72
*Nicotiana tabacum*	155.8	25.3	18.4	86.7	98	45	75
*Vitis vinifera*	160.9	26.6	19.1	89.1	84	53	16
*Phaseolus vulgaris*	150.3	26.4	17.6	79.8	83	44	10
*Triticum aestivum*	134.5	20.7	12.8	80.3	83	50	63
*Pinus thunbergii*	119.7	0.6	53.0	65.7	123	39	nd
*Zea mays*	140.4	22.7	12.5	82.3	111	46	nd
*Chlamydomonas reinhardtii*	203.4	22.2	78.1	80.9	69	40	nd
*Dunaliella salina*	269.0	14.4	112.9	127.3	82	36	nd
*Ostreococcus tauri*	71.7	6.8	22.3	35.9	61	33	nd
*Helicosporidium* sp. *ex Simulium*	37.5	ab	ab	ab	26	28	nd
*Euglena gracilis*	143.2	ab	ab	ab	67	48	399
*Nephroselmis olivacea*	200.8	46.1	16.4	92.1	155	45	ab
*Gracilaria tenuistipitata*	183.8	ab	ab	ab	203	33	nd

All data comes from NCBI and the Chloroplast Genome Database

*IR* inverted repeats, *SSC* small single copy, *LSC* large single copy, *ab* absence, *nd* no data

Typical plastomes have a pair of identical inverted repeats (IRs), 5–76 kbp each (although in *Pinus thunbergii* they are just 600 bp long), separated by small and large single copy regions (SSC and LSC, respectively) (Fig. 1). IRs usually contain three highly conservative regions coding rRNAs and some tRNAs and are very similar to each other. Exceptions from this rule were discovered in *Pisum sativum*, *Vicia faba*, *Medicago sativa* (which contains a single IR-like sequence), and *Euglena gracilis* (3 direct repeats). Some plastomes do not have inverted repeats (e.g., *Helicosporidium* sp. *ex Simulium*, *Euglena gracilis*, *Gracilaria tenuistipitata*).

Sequences separating IRs, short and long single copy regions, are unique. Size of the latter has major influence on size of the entire plastome (Shaw et al. 2007). One of the largest LSCs (127.3 kbp) has been found in *Dunaliella salina* (Table 1).

Plastome usually contains 100–120 functional genes, and their size does not directly correlate with plastome size. All chloroplast genomes contain genes encoding proteins, tRNA and rRNA (De Las Rivas et al. 2002; Cui et al. 2006; Wicke et al. 2011). Among species with more genes, some genes (mostly protein-encoding ones) still need to be analyzed. For example, *Chlorella vulgaris* is estimated to have 174 genes (including 63 of hypothetical or uncharacterized proteins) (Cui et al. 2006). Vast majority of telomic plants has 80–100 defined protein coding genes (and only a few undefined, whose products participate in: translation, transcription, photosynthesis, energy metabolism, fatty acid metabolism, transport, and cofactor biosynthesis (Barkan 2011; Green 2011). RNA genes are defined more accurately—6–8 rRNA genes are present in IRs, and 36–40 tRNA genes are spread across the entire plastome (Wicke et al. 2011).

Aside from aforementioned genes, many conserved hypothetical ORFs (ycfs) were discovered, but their functions remained unknown until recent years. For a long time, *ycf1* gene remained the biggest mystery—while its presence was necessary for survival of researched organisms (*Nicotiana tabaccum* and *Chlamydomonas reinhardtii*), its function was unknown, and its absence in plastomes of *Poaceae* posed more questions (Boudreau et al. 1997; Drescher et al. 2000; Guisinger et al. 2010; de Vries et al. 2015). However, in 2013, it has been discovered that in *Arabidopsis thaliana,* this gene encodes Tic214, which is a part of general TIC translocon, necessary for transfer of nuclear proteins into the chloroplast (Kikuchi et al. 2013). Currently, *ycf2* remains as the only hypothetical ORF whose protein has an unknown function.

An interesting parameter of plastomes is their number of introns. In land plant plastomes, group I and II introns have been identified (with the latter representing over 90 % of intron pool) (Eckard 2007). However, plastomes of genus *Euglena* also contain 73–119 nt long group III introns, derived from group II introns, but lacking their D2–D5 domains (Sheveleva and Hallick 2004). Introns are present both in protein-coding and RNA genes.

Among selected species representing the monocots, the intron number is as follows: *Oryza sativa*—60; *Hordeum vulgare*—14; *Triticum aestivum*—63; among dicots: *Vitis vinifera*—16; *Solanum tuberosum*—22; *Nicotiana tabacum*—75; *Populus trichocarpa*—26; *Pinus koraiensis*—36; and *Arabidopsis thaliana*—72 (Cui et al. 2006). *Euglena gracilis* has a distinctively high number of introns (399—over 40 % of total ctDNA). Its special trait is the presence of 15 twintrons—group II and III introns present within other introns from these groups. Simple and complex twintrons can be distinguished—in the former, a single intron is inserted into another, while the representatives of the latter group consist of at least two introns inserted into another intron (Hallick et al. 1993).

However, in most of species, the number of introns varies between one and few dozen, with no regularity among species from the same taxon—Solanaceae (*Solanum tuberosum*—22, *Nicotiana tabacum*—75, and *Lycopersicon esculentum cultivar LA3023*—17) and Fabaceae (*Lotus corniculatus*—69, *Glycine max*—25, and *Phaseolus vulgaris*—10) are a prime example. On the other hand, some species have no introns whatsoever (*Cyanidioschyzon merolae*, *Cyanidium caldarium*, *Gracilaria tenuistipitata*, *Nephroselmis olivacea*, and *Porphyra purpurea*) (Cui et al. 2006).

A different content of GC pairs is a trait strongly distinguishing plastomes from nuclear genomes. In former, GC pairs represent just ~30 % of total base pairs—it is caused by multiple traits of chloroplast, such as specific properties of chloroplast DNA polymerase and repair systems (Nielsen et al. 2010).

## Chloroplast gene expression

### Transcription: PEP and NEP polymerases

Despite heavily depending on nucleus-derived proteins, chloroplasts possess their own system responsible for expression of genes, which originate from ancestral cyanobacteria (Yagi and Shiina 2014). RNA polymerases, responsible for transcription, are a crucial part of this machinery. Two types of them can be found in a chloroplast—bacterial-type plastid-encoded RNA polymerase known as PEP and nuclear-encoded RNA polymerase (NEP), characteristic for angiosperms and *Physcomitrella* moss (Barkan 2011, Yagi and Shiina 2014, Liere et al. 2011). PEP consists of four subunits, α-, β-, β′-, and β″, which are encoded by *rpoA*, *rpoB*, *rpoC1*, and *rpoC2* plastid genes, respectively (Serino and Maliga 1998). It shares multiple traits, such as need for sigma factors in promoter recognition, with eubacterial RNA polymerases (however, it is worth noting that plant sigma factors are encoded in nucleus) (Tiller and Bock 2014). There are multiple types of sigma factors, which grant promoter specificity and take part in transcription of different groups of genes (Lerbs-Mache 2011; Yagi and Shiina 2014). Meanwhile, NEP is a single-subunit polymerase, which shares many similarities with its counterparts in mitochondria and T3/7-type bacteriophage (Liere et al. 2011). Chloroplasts of higher plants contain two types of NEP (RPOTp and RPOTmp), which are capable of aiding PEP with transcription of plastome genes (Swiatecka-Hagenbruch et al. 2008).

PEP and NEP also recognize different promoters. PEP promoters usually contain consensus sequences, −35 (TTGaca) and −10 (TAtaaT), and resemble bacterial σ70 promoters (Gruissem and Zurawski 1985; Yagi and Shiina 2014). On the other hand, NEP has two main types of promoters—type I has an YRTA motif, which bears many similarities to mitochondrial promoters, and sequences crucial for type II promoters are placed downstream of the transcription initiation site (Sriraman et al. 1998; Swiatecka-Hagenbruch et al. 2008).

### Post-transcriptional processing and translation

After transcription, initial transcripts undergo a multi-step processing, which includes splicing, transforming primary RNA molecules into mono- or oligocistronic mRNAs, formation and maturation of 5′ and 3′ ends, and finally RNA editing (Stern et al. 2010; Tiller and Bock 2014). Most of components of translation in plastids (including 70S ribosomes) have cyanobacterial homologues (Tiller and Bock 2014). However, plastid translation also involves plenty of protein translation factors and positive regulatory mRNA elements, which do not have bacterial counterparts and increase the overall complexity of the process (Manuell et al. 2007).

The process usually is initiated by small subunit (30S) of the ribosome and transfer RNA (tRNA), which selects the initiation site on the 5′ end of mRNA (usually starting from AUG triplet, although in some cases, GUG and UUG can also serve that role) (Sugiura et al. 1998). Once the site is selected, 50S subunit is attached to the complex, resulting in activation of initiation complex and start of elongation phase (Manuell et al. 2007). During this stage, ribosome progresses toward 3′ end of mRNA and adds amino acids carried by tRNA to the growing polypeptide. This process is supported by translation elongation factors: eEF1A, eEF1B, eEF2, and eEF5 (Doerfel et al. 2013; Gutierrez et al. 2013; Browning and Bailey-Serres 2015). Each of these factors has a different purpose: eEF1A is responsible for delivering tRNA to the peptidyl transferase center, eEF1B serves as exchange factor for eEF1A in recycling GDP for GTP, eEF2 plays a role in moving tRNA that already dropped its amino acid off from P-site to E-site and eEF5 increases the efficacy of proline and glycine-rich protein elongation (Browning and Bailey-Serres 2015).

Termination of translation is caused by the ribosome reaching one of STOP codons (UAA, UAG, UGA). Once it happens, two release factors (eRF1 and eRF3) bind the STOP codon and cut the newly created peptide off, paving the way for recycling of the ribosome (Dever and Green 2012).

## Plastid genome sequencing

The research focused on learning the primary structure of plastomes has begun significantly earlier than similar studies regarding nuclear genomes. First, complete chloroplast genome sequences were published in second half of 1980s (Shinozaki et al. 1986), while the analogous data about eukaryotic nuclear genomes and prokaryotic genomes emerged over a decade later. It was caused by a major difference in genome size and the level of advancement of sequencing techniques. These two factors are the main reason for current abundance of knowledge about structure and functioning of plastomes. At first, it had a purely cognitive character, but with time, it also gained a major applicational importance. Referential databases (such as NCBI) currently contain information about chloroplast DNA of over 1000 plant species. Most of them represent *Viridiplantae*—a clade of green plants, containing Chlorophyta and telome plants (Cocquyt et al. 2009). However, representatives of other taxons can also be found there, providing multiple opportunities of performing multi-directional comparative analyses. Knowledge regarding organization and mechanisms of functioning of these genomes has drawn increasing attention of scientists. This interest is expressed in form of constantly growing number of analyzed species. Plastomes became an attractive target of intentional modifications, whose purpose is utilizing their capabilities in many fields of modern biotechnology—this trend is reflected by the amount of realized sequencing projects. Genome engineering includes transfer of new genes (or other specific elements, such as promoters) and changes aimed at controlling expression of endogenous genes. Creation of new construct and precise control of results is easier in plastomes than in nuclear genome. The following chapters describe selected aspects of plastome modification.

## Plastid genome engineering

In recent years, plastid transformation has emerged as an attractive alternative for nuclear gene transformation. However, the specific nature of chloroplasts posed multiple challenges in designing techniques that could be utilized in this process.

### Designing plastid transformation

The first and biggest obstacle was selecting DNA vectors. Contrary to a random, *Agrobacterium*-mediated T-DNA integration in nuclear transgenes, chloroplast transgene integration is targeted due to homologous recombination of vector’s flanking sequences and target DNA (Verma and Daniell 2007; Meyers et al. 2010). Because of that, transgene must be flanked by two targeting sequences, each around 1–2 kb in size (Meyers et al. 2010). Currently, *Escherichia coli* plasmids incapable of replication in plastids are the most popular vectors. However, maintaining efficiency of transplastomic plant generation requires reconstructing the vectors when a different plant species is chosen (Verma and Daniell 2007). Depending on the state of a target, DNA is introduced either via polyethylene glycol treatment (when protoplasts are targeted) or gene gun (when plastids are in an undamaged tissue) (Dix and Kavanagh 1995; Maliga and Bock 2011). Selection of transformed plants is usually done by utilizing marker genes encoding resistance to antibiotics. Currently, the most popular choice is *aadA*, a gene from *E. coli* responsible for encoding aminoglycoside 3″-adenylyl transferase, which grants resistance to spectinomycin (Bock 2014a).

It must be remembered that usually no more than a few plastomes undergo transformation. To prevent the subsequent loss of transplastomes, propagation of transplastomic cell lines under selection pressure has to be maintained until all the wild-type genomes perish (Bock 2014b).

### Plastid engineering: pros and cons

Plastid engineering holds some important advantages over its nuclear counterpart. First, vast majority of angiosperms inherits chloroplasts maternally, thus significantly reducing the risk of transgenic plants outcrossing with weeds via pollen—in turn, reduction of that risk means that transgene silencing, utilized in commercially used transgenic plants to prevent outcrossing, is no longer necessary (Wani et al. 2010). Transformed plastids are also capable of producing a significantly higher amount of target protein, reaching from 4 to 46.1 % (or, in most extreme cases, over 70 %) of total soluble protein (TSP), compared with 1–2 % in plants where nuclear genome underwent transformation (De Cosa et al. 2001; Daniell 2006; Oey et al. 2009; Meyers et al. 2010). Usefulness of plastome transformation is further boosted by lack of silencing mechanisms which can interfere with the process and the ability to transfer full metabolic pathways into the plastome while maintaining their efficiency (Meyers et al. 2010).

While plastome engineering seems promising, it is important to remember that this technique is not free of limitations. Most importantly, multiple economically important plants lack feasible protocols for plastid transformation; the alternative is to transfer transgenic plastid from transformable plants to the species of choice (Sigeno et al. 2009; Ovcharenko et al. 2011). Unfortunately, this method is costly and time-consuming, which significantly limits its application in comparison to nuclear transformation. However, it has been discovered that movement of cpDNA between cells of grafted plants is possible and can potentially improve efficiency of the whole process (however, it happens at a cost of increased likelihood of mutations and is limited to closely related species) (Bock 2014a). It is also worth noting that proteins do not undergo glycosylation in transformed chloroplasts, although the impact of lack of this process depends on the protein of interest (McCabe et al. 2008).

## Potential use of plastome analysis and modification

Thanks to significant progress in plastome research, a potential range of ways to utilize plastome analysis and modification has greatly increased. Here, we present the most promising types of current plastome research.

### Production of antigens, vaccines, and therapeutic proteins

One of the most promising potential uses of plastome engineering is the production of recombinant proteins. High yield of target protein in transformed plastids and, among many positive traits, ability to apply post-translational modifications combined with lack of silencing, makes using chloroplast modification for production of antigens, enzymes, therapeutic proteins and vaccines, and intriguing prospect.

Over the last 15 years, we have witnessed the emergence of multiple reports on production of bacterial and viral antigens that can be used in vaccines directed against the most dangerous human diseases. However, most of them have only been tested on mice so far, which means that developing effective vaccines for humans will still take time (Daniell et al. 2009). Initially, most of vaccine antigens were expressed in tobacco leaves. The first antigen expressed in tobacco was the cholera toxin B subunit (CTB), whose accumulation reached 4.1 % of total soluble leaf protein (TLP) (Daniell et al. 2001a). Fusing CTB with merozoite surface protein-1 (MSP1) and apical membrane antigen-1 (AMA1) further increased TSP values (to 10.11 and 13.17 %, respectively) and isolated antigens provided inoculated mice with long-time protection against *Vibrio cholerae* (Davoodi-Semiromi et al. 2010). Other bacterial antigens successfully accumulated in tobacco include: heat-labile enterotoxin subunit B of *E. coli* responsible for diarrhea (Kang et al. 2003), tetanus toxin fragment C (Tregoning et al. 2005), and anthrax protective antigen (Watson et al. 2004; Ruhlman et al. 2007).

Tobacco has also proved to be a promising platform for production of viral antigens. In 2004, rotavirus VP6 protein was successfully accumulated in seedlings and young leaves of tobacco, reaching 3 % TSP, but it lacked stability and was never tested on animals (Birch-Machin et al. 2004). Since then, most of research focused on antigens of three viruses—cervical cancer-causing human papillomavirus (HPV), human immunodeficiency virus (HIV), and hepatitis virus. Eventually, antigens derived from all of these viruses were produced in tobacco. As it turned out, the target proteins maintained their effectiveness—human papillomavirus antigens (L1 and E7) induced systemic immune response in mice (Fernández-San Millán et al. 2008; Venuti et al. 2015), and so did p24 and C4V3 polypeptides isolated from HIV, which successfully elicited antibody responses in mice (Gonzalez-Rabade et al. 2011; Rubio-Infante et al. 2012).

While the yield and stability of target proteins were satisfactory, tobacco’s usefulness was limited by its high alkaloid content, which made it unsuitable for oral delivery. Because of that, scientists have searched for more suitable plants for antigen accumulation, and they have found them in form of lettuce and tomatoes. Both of them turned out to be effective for production and oral administration of target proteins, although it is worth noting that ripening of tomatoes reduced their transgene expression (Zhou et al. 2008; Davoodi-Semiromi et al. 2010; Lössl and Waheed 2011). Research on lettuce already resulted in successful production of multiple important therapeutic proteins; the first successful attempt was accumulating proinsulin, where old leaves of plants with chloroplasts transformed with human proinsulin–CTB complex managed to accumulate proinsulin up to 53 % of TLF, compared with 47 % in old tobacco leaves and just 1 % of total seed protein in *Arabidopsis* (Boothe et al. 2010; Boyhan and Daniell 2011). Some algae have also proved to be capable of producing vaccine antigens—in 2010 *Chlamydomonas reinhardtii* was successfully utilized to accumulate AMA1 and MSP1, two potential candidates for vaccine against malaria-causing protozoa from *Plasmodium* genus (Dauvillée et al. 2010).

### Enzyme production: obtaining biomass and raw material

Transplastomes can also be used to synthesize enzymes, which can be utilized to obtain material for biofuel. Currently, biofuel production is an expensive process, which results in wasting of large amounts of potentially useful biomaterial. Big part of expenses comes from obtaining enzymes necessary for degradation of cell walls—cellulases, hemicellulases, and accessory enzymes (Agrawal et al. 2011). That is why in recent year’s utilization of modified plants as a source of enzymes became a popular field of research. So far, none of the enzymes derived from transgenic plants are produced on a mass scale, but current results show immense promise. For example, endoglucanase (cellulase) and pectate lyases (hemicellulases) from one of experimental cultivars of transplastomic tobacco were significantly less expensive than commercial enzymes (production was 3100-fold and 1057/1480-fold cheaper, respectively) (Verma et al. 2010).

Another enzyme expressed in transformed tobacco chloroplasts is β-mannanase, a hemicellulase of *Trichoderma reesei.* Once again, the results gave reasons to be optimistic—mannase produced in chloroplasts had wider pH optima and thermostability than its counterpart obtained from *E. coli* and displayed notable activity even without purification, potentially opening a route for its easier and cheaper utilization in the future (Agrawal et al. 2011). Another examples of fungal enzymes successfully obtained from transplastomic tobacco are swollenin and cutinase; however, it is unlikely for them to find any practical use soon, as they caused severe damage to plant cells—it included damaged thylakoid membranes, disruption of pigment-protein complexes, loss of chloroplasts, and decreased galactolipids content (Verma et al. 2013). Tobacco chloroplasts are also capable of producing bacterial enzymes, such as thermostable cellulases (Cel6A and Cel6B) of thermophilic actinobacteria, *Thermobifida fusca* (Yu et al. 2007).

### Crop improvement

Every plant is exposed to a variety of biotic and abiotic stressors, which are capable of negatively impacting plant’s development, size, and quality of its yield. Because of that, plastome modifications introducing improved resistance to common stress factors are a popular field of research, which has potential to improve the yield, reduce the amount of used pesticides and insecticides, and give a chance to grow the crop in areas where wild-type plants would struggle to survive.

#### Herbicide resistance

The increasing use of herbicides has resulted in the need for plants capable of surviving exposure to non-selective chemicals used against the weeds. However, providing it by nuclear transformation of crops is a risky approach, as herbicide immunity genes can be transferred with pollen, potentially resulting in outcrossing with weeds and subsequent creation of superweeds, immune to herbicides. While it is possible to significantly reduce that threat by silencing transgenes, this process further increases the cost of the whole operation. This is why chloroplast transformation, free of risk coming with outcrossing, is being looked into.

First successes in providing herbicide resistance through genetic engineering of plastome came in 1998. The research focused on finding a way to reduce or remove the negative influence of glyphosate. This objective was achieved by transferring to tobacco’s chloroplast the petunia gene engineered to overexpress glyphosate’s target, 5-enol-pyruvyl shikimate-3-phosphate synthase (EPSPS), catalyst of one of the key steps of aromatic amino acid biosynthesis, (Daniell et al. 1998). In the following years, it has been shown that prokaryotic EPSPS can also be successfully used in this procedure, although its accumulation and glyphosate tolerance did not correlate (Ye et al. 2001).

Another example of successful induction of herbicide resistance includes improving resistance to some of the triketone herbicides by introducing barley’s 4-hydroxyphenylpyruvate dioxygenase-synthesizing *hppd* gene, which is a part of plastoquinone and vitamin E biosynthesis pathway, into tobacco plastomes (Falk et al. 2005; Venkatesh and Park 2012). In 2007, *hpdd* from *Pseudomonas fluorescens* was introduced into soybean plastomes; this experiment is especially noteworthy, as it was the first case of obtaining herbicide immunity in an economically important crop without utilizing antibiotic resistance markers (Dufourmantel et al. 2007).

#### Pest and disease resistance

Pests and diseases are some of the biggest problems for modern agriculture. Their influence is capable of severely reducing the crop yield and forces the use of chemicals, which represent an environmental hazard and increase the overall cost of crop growth. While nuclear transformation has managed to reduce the impact of these factors, chloroplast transformation can still provide additional biosafety and play a role when higher gene expression is needed (Venkatesh and Park 2012).

The first discoveries in this field were made in 1995, when crylA(c) coding sequence of *Bacillus thuringiensis* (Bt) was used to produce Bt toxins in tobacco in concentrations which made it harmful for attacking insects, such as *Helicoverpa zea*, *Heliothis virescens,* and *Spodoptera exigua* larvae (McBride et al. 1995). Since then, Bt toxins were introduced into other transplastomic plants, such as cabbage (*Brassica oleracea*), but they never entered commercial production, mainly due to plenty of available alternatives obtained by nuclear tranformation (Liu et al. 2008; Jin and Daniell 2015).

Disease-resistant tobacco was first created in 2001 by inserting the sequence encoding an antimicrobial peptide MSI-99, analog of magianin 2, defensive peptide secreted from the skin of *Xenopus laevis* (African clawed frog) (DeGray et al. 2001). Transplastomic plants significantly inhibited growth of pre-germinated *Aspergillus flavus*, *Fusarium moniliforme*, and *Verticillium dahlia* fungi, and *Pseudomonas syringae* pv. tabaci bacteria (DeGray et al. 2001). Recent research also shows that MSI-99 expressed by tobacco chloroplasts is capable of suppressing the effects of rice blast, one of the most dangerous fungal rice diseases (Wang et al. 2015).

#### Simultaneous resistance to herbcides, pests, and diseases

In recent years, a number of transplastomic plants capable of simultaneously improving resistance to insects and diseases have emerged. While this kind of immunity would usually require multi-gene engineering, transforming tobacco chloroplasts with agglutinin gene from *Pinellia ternata,* a popular Chinese medicinal herb, is enough to singlehandedly reduce the survival rate of a wide array of pests (*Bemisia tabaci* whitefly, *Myzus persicae* aphid, *Spodoptera exigua* armyworm, *Helicoverpa zea*, *Heliothis virescens,* and *Lepidopteran* insects) by 90–100 %; also, agglutinin displayed significant anti-viral activity (Jin et al. 2012). Another example of transplastomic plant immune to insects and phytopathogens is *Nicotiana benthamiana* whose plastids included genes encoding chitinase from *Paecilomyces javanicus*, sporamin from sweet potato and cystatin from *Colocasia esculenta* (Chen et al. 2014). Bioassays have confirmed that the transformation reduced the symptoms of soft rot caused by *Pectobacterium carotovorum* subsp. *carotovorum* and leaf spot disease associated with *Alternaria alternata* and caused growth retardation and death of *Spodoptera exigua* and *S. litura* larvae that ingested the leaves (Chen et al. 2014).

#### Abiotic stress resistance

Growing demand for plants capable of growing and giving yield in unfavorable conditions, combined with human exploitation and extreme weather phenomena lead to an increased need for plants with increased immunity to drought, extreme temperatures and salinity.

To increase immunity to salt stress and drought, osmoprotectants are necessary due to their role in stabilizing membranes and proteins during osmotic stress. As most of plants cannot metabolize sugar alcohols, introducing genes encoding a product capable of that is a viable way to improve osmoprotection (Khan et al. 2015). One of such genes is *ArDH*, which encodes arabitol dehydrogenase, responsible for reduction of d-ribulose to d-arabitol, which accumulates in chloroplasts and increases tolerance to high NaCl concentrations. Its expression in tobacco chloroplasts allows plants to grow normally on soil with up to 350-mM NaCl concentration (Khan et al. 2015). Another example is a betaine dehydrogenase (BADH) gene transformed into *Daucus carota* (carrot), which resulted in transformed plants accumulating 50- to 54-fold more betalaine than their untransformed counterparts in medium with 100-mM NaCl concentration (Kumar et al., 2004). Transplastomic *N. benthamiana* with genes encoding chitinase, sporamin, and cystatin, mentioned before due to its increased immunity to insects and diseases, also shows increased immunity to osmotic stress (Chen et al. 2014). Meanwhile, temperature stress resistance can be achieved by enhancing antioxidant defense, increasing the unsaturation of fatty acids or by the use of *E. colipanD* gene encoding l-aspartate-α-decarboxylase—the enzyme decarboxylating l-aspartate to β-alanine and carbon dioxide (Wani et al. 2015).

## Conclusions

Once the most important issues related to mass cultivation of tranplastomic plants are solved, they are likely to become a viable alternative for plants with nuclear transgenes, because of their higher production of protein of interest, lack of silencing mechanism, significantly higher biosafety, ability to engineer entire metabolic pathways, freedom of applying desired posttranslational modifications to the protein of interest, and potentially, no need for purification of protein target. At the moment, it is unclear whether transplastomic plants can become more important for agriculture or biopharmaceutics, as we still failed to get anywhere close to uncovering their real potential. However, it is worth remembering that there are some exciting subjects related to both worlds, such as edible vaccines. For this reason, cooperation between scientists, agricultural and biotechnological companies will be necessary for steady development of transplastomics.

Currently, the biggest challenges ahead are related to the lack of protocols for transforming plastomes of other plants than tobacco. While attempts to express foreign genes in some important agricultural plants, such as lettuce and tomato, have already succeeded, their number is still too low, the protocols contain many flaws, and the need to create a separate protocol for every cultivar makes the whole process more expensive. On top of that, there are still no successful attempts of creating transplastomic staple food crops, such as cereal, and expressing foreign genes in non-green plastids remains a challenge due to our poor understanding of their genetic structure and expression (Wani et al. 2015). It is very unlikely for any transplastomic plants to be commercially used in the near future due to the reasons mentioned above combined with low plant regeneration efficiency and widespread use of antibiotic resistance genes as markers. At this time, it appears that the problem of markers is the closest to the solution, as safer, although not widespread markers unrelated to antibiotic resistance already exist and some of them, such as *BADH*, are even capable of improving the target plant (Daniell et al. 2001b).

Information about plastome structure can also be very valuable in phylogenetic analyses and is an efficient molecular tool due to low mutation rate, easy amplification and conservative sequences of chloroplast genome (Hu et al. 2015). To this day, plastomes have been used in multiple analyses on different taxonomy levels—from species of the same genus to clades (Moore et al. 2007, 2010, Hu et al. 2015). Until now, the biggest issue was a low number of sequenced plastomes, which negatively affected the quality of phylogenetic analysis (Rogalski et al. 2015). However, recent major progress in realization of such projects means that the aforementioned analyses have a chance to become more reliable.

### *Author contribution statement*

SO, EŁ, TK and TS wrote the manuscript. EŁ provided the tables and figures. SO did final revisions of the manuscript. All authors read and approved this manuscript.
